# Editorial: Psychiatric illness across the menstrual cycle

**DOI:** 10.3389/fpsyt.2025.1568088

**Published:** 2025-02-12

**Authors:** Liisa Hantsoo, Lauren M. Osborne, Leah C. Susser

**Affiliations:** ^1^ Department of Psychiatry & Behavioral Sciences, The Johns Hopkins University School of Medicine, Baltimore, MD, United States; ^2^ Department of Obstetrics & Gynecology, Weill Cornell Medical College, New York, NY, United States; ^3^ Department of Psychiatry, Weill Cornell Medical College, New York, NY, United States; ^4^ Department of Psychiatry, Weill Cornell Medicine, White Plains, NY, United States

**Keywords:** premenstrual, menstrual cycle, PMDD, PMS, premenstrual exacerbation (PME), women’s health

Premenstrual psychiatric symptoms are common and occur in several disorders including premenstrual dysphoric disorder (PMDD), premenstrual syndrome (PMS), and premenstrual exacerbation (PME) of psychiatric illness. In PMDD and PMS, symptoms are restricted to the luteal phase of the menstrual cycle; in PME, symptoms of an underlying affective disorder are exacerbated in the luteal phase. PMDD is a severe mood disorder characterized by affective symptoms with onset 1-2 weeks before menses and offset once menstruation begins, or shortly thereafter (1). In contrast, PMS is a broader term encompassing fewer and less impairing symptoms (2), and can be diagnosed based on physical symptoms *or* affective symptoms. Common affective symptoms in PMDD and PMS include irritability, low mood, anxiety, sensitivity to stressors, and mood lability. PME is the cyclic premenstrual worsening of a psychiatric disorder, such as major depressive disorder, in which existing symptoms become more severe in the week before menses, then return to an elevated baseline following menses onset or resolution (3). In PME, unlike PMDD and PMS, symptoms do not resolve in the follicular phase of the menstrual cycle.

Despite the associated prevalence and impairment, premenstrual disorders remain understudied relative to other psychiatric disorders, and even relative to other reproductive affective disorders such as postpartum depression (4). This Research Topic aims to increase awareness of menstrual cycle-related psychiatric conditions, to promote research in this understudied field, and to improve clinical recognition. Articles in this Research Topic focus on: 1) understanding clinical features and treatment of premenstrual disorders, 2) understanding the prevalence of PME across psychiatric disorders, 3) identifying risk factors for premenstrual disorders, and 4) predicting individual symptom onset in the menstrual cycle, which may enable rapid intervention.

Several articles in this Research Topic focus on clinical features and treatment of premenstrual disorders. An article by Brown D. et al. reviews the qualitative psychological experience of PMDs (PMS and PMDD). The authors highlight the distress and impairment women experience from PMDs and the limited information they receive to manage their symptoms, often requiring them to advocate for themselves in the healthcare setting and devise their own coping strategies, including both adaptive and maladaptive approaches. Premenstrual symptoms affected them across multiple domains of their lives, including relationships and career. Importantly, a research paper in this Research Topic, also by Brown D. et al., finds that among women with PMDD surveyed, nearly half had deliberately harmed themselves during a PMDD crisis, 82% reported premenstrual suicidal ideation, and 26% had attempted suicide. SI was influenced by personal relationships affected by PMDD, diagnosis delays, and self-worth damaged by PMDD. This high rate of self-harm and suicidality emphasizes the seriousness of this disorder. Within reproductive mental health, gender-diverse individuals are particularly understudied and underserved. An article by Arshed et al. focuses on transgender and gender-diverse (TGD) menstruators. This population’s experience of menstruation and associated mood symptoms is unique and deserves special consideration by clinicians and researchers. The authors provide suggestions for the psychiatric management of menstruation in TGD and promote gender-affirming menstrual care for transmenstruators. In addition, Islas-Preciado et al. review cultural attitudes and taboos toward premenstrual symptoms, and emphasize the need for an intersectional approach that acknowledges interacting social identities such as race, gender, and sexuality that may influence women’s experiences of premenstrual mood symptoms. Modzelewski et al. explore treatment of premenstrual mood symptoms in general, including SSRIs, hormonal agents, and therapy.

PME affects a large number of women living with psychiatric illness. Lin et al. review PME of mood, anxiety, psychotic, obsessive-compulsive, personality, and trauma-related disorders. The authors review data on PME prevalence and describe treatment options. They note that there is little guidance on assessment of PME, resulting in a paucity of research and clinical recommendations. Guidelines and clinical tools for assessment of PME are needed, and awareness of PME in clinical practice is imperative for diagnosis and alleviation of associated distress and functional impairment. For those who require medication, treatment for PME differs from that for PMDD – so it is vital for clinicians to understand the distinction.

Risk factors for premenstrual disorders are a growing area of study. Adverse childhood experiences are known to increase risk for PMDD and PMS (5), but less is known about PME. A study by Standeven et al. finds that individuals with PME have a higher quantity and severity of childhood traumatic events compared to healthy controls, with a positive correlation between childhood trauma and premenstrual symptom burden.

While premenstrual disorders remain underrecognized and understudied, advances in research on premenstrual psychiatric symptoms are on the horizon. Brown R. et al. describe how wearable technologies may improve measures of physiologic features such as heart rate variability, sleep, and physical activity, to advance research in this area. They suggest that, in the future, remote digital monitoring paradigms may enable patients and physicians to monitor and respond to premenstrual symptoms in real-time. This would allow women to recognize and target premenstrual symptoms before they interfere in functioning, lead to harmful suicidal and non-suicidal behaviors, or harm interpersonal relationships. The articles discussed previously describe the distress and consequences of premenstrual disorders, demonstrating the urgent need for real-time detection and treatment. Relatedly, Tauseef et al. discuss possibilities for just-in-time adaptive interventions (JITAIs) to target premenstrual psychiatric symptoms, including affective dysregulation, irritability, and suicidality. They propose that JITAIs could use menstrual cycle data to identify points of vulnerability within individuals and strategically deploy interventions based upon their individual profile. This would provide a personalized medicine approach to managing premenstrual symptoms.

Together, this Research Topic suggests that, while research on psychiatric illness across the menstrual cycle is limited, it is a quickly growing area of research, with exciting horizons ahead that may include better characterization of risk factors, improved treatments based on a more advanced understanding of the biological features of premenstrual disorders, and use of cutting-edge technology to manage premenstrual disorders. Improvements in identification of at-risk populations, detection of symptom onset in individuals, and treatment can reduce the burden of premenstrual disorders on many women’s lives.

## References

[1] American Psychiatric Assn A. Diagnostic and statistical manual of mental disorders (5th ed., text rev.). 5th ed. Arlington VA: American Psychiatric Publishing (2022).

[2] ACOG. Management of premenstrual disorders. ACOG Clinical Practice Guideline No. 7. Int J Obstet Gynecol. (2023) 142:1516–33. doi: 10.1097/AOG.0000000000005426 37973069

[3] Kuehner C Nayman S. Premenstrual exacerbations of mood disorders: findings and knowledge gaps. Curr Psychiatry Rep. (2021) 23:78. doi: 10.1007/s11920-021-01286-0 34626258 PMC8502143

[4] Hantsoo L Payne JL. Premenstrual dysphoric disorder affects five times as many individuals as postpartum depression, but receives 80% Less NIH research funding. J Womens Health (Larchmt). (2024) 33:1296–7. doi: 10.1089/jwh.2024.0787 39034897

[5] Hantsoo L Jagodnik KM Novick AM Baweja R di Scalea TL Ozerdem A. The role of the hypothalamic-pituitary-adrenal axis in depression across the female reproductive lifecycle: current knowledge and future directions. Front Endocrinol (Lausanne). (2023) 14:1295261. doi: 10.3389/fendo.2023.1295261 38149098 PMC10750128

